# Supplementary material for: A UMLS-based spell checker for natural language processing in vaccine safety
Source: BMC Med Inform Decis Mak. 2007 Feb 12;7:3. doi: 10.1186/1472-6947-7-3 (PMC1805499; doi:10.1186/1472-6947-7-3)
Supplement: Additional file 13 — RAP application directory. Sets up directory for RAP (RDF application for PHP) source code [file 1472-6947-7-3-S13.gz › rap/doc/database_schema.html]

Test Set Database Connection


### RDF API for PHP V0.9.1

# Database Schema

##### RAP uses a denormalized database layout for increased performance.

  
  
  

## Table models

|  |  |  |
| --- | --- | --- |
| **Column name** | **Type** | **Comments** |
| modelID | id-ref, indexed |  |
| modelURI | varchar | used at reopening a persistent model |
| baseURI | varchar | affects creating of new resources and serialization syntax |

  
  
  

## Table statements

|  |  |  |
| --- | --- | --- |
| **Column name** | **Type** | **Comments** |
| modelID | id-ref, indexed |  |
| subject | varchar, indexed |  |
| predicate | varchar, indexed |  |
| object | blob, indexed |  |
| l\_language | varchar | only if object is a literal, otherwise NULL |
| l\_datatype | varchar | only if object is a literal, otherwise NULL |
| subject\_is | varchar(1) | flags whether subject is a resource or blankNode |
| object\_is | varchar(1) | flags whether object is a resource, blankNode or literal |

  
  
  

## Table namespaces

|  |  |  |
| --- | --- | --- |
| **Column name** | **Type** | **Comments** |
| modelID | id-ref, indexed |  |
| namespace | varchar |  |
| prefix | varchar |  |

##### For best performance we recommend joint indexing on subject and predicate.

## Table datasets

|  |  |  |
| --- | --- | --- |
| **Column name** | **Type** | **Comments** |
| datasetName | varchar |  |
| defaultModelUri | varchar |  |

## Table dataset\_model

|  |  |  |
| --- | --- | --- |
| **Column name** | **Type** | **Comments** |
| datasetName | varchar |  |
| modelID | int |  |
| graphURI | varchar |  |
